# Vaginal McCall culdoplasty versus laparoscopic uterosacral plication to prophylactically address vaginal vault prolapse

**DOI:** 10.1186/s10397-017-1006-4

**Published:** 2017-04-12

**Authors:** Kathy Niblock, Emily Bailie, Geoff McCracken, Keith Johnston

**Affiliations:** 1 Craigavon Area Hospital, 68 Lurgan Rd, Portadown, Craigavon, BT63 5QQ Northern Ireland; 2 Antrim Area Hospital, 45 Bush Rd, Antrim, BT41 2RL Northern Ireland

**Keywords:** Vault prolapse, McCall culdoplasty, Uterosacral plication

## Abstract

**Background:**

Studies have shown that vaginal vault prolapse can affect up to 43% of women following hysterectomy for pelvic organ prolapse. Many techniques have been described to prevent and treat vaginal vault prolapse. The primary objective of our study was to compare McCall’s culdoplasty (when performed along side vaginal hysterectomy) with laparoscopic uterosacral plication (when performed along side total laparoscopic hysterectomy) for prevention of vaginal vault prolapse. Secondary outcomes included inpatient stay and perioperative complications.

A retrospective comparison study comparing 73 patients who underwent ‘laparoscopic hysterectomy and uterosacral plication’ against 70 patients who underwent ‘vaginal hysterectomy and McCall culdoplasty’. All operations were carried out by two trained surgeons.

**Results:**

There was no significant difference between BMI or parity. There were statistically significantly more patients presenting with post hysterectomy vault prolapse (PHVP) in the group of patients who had undergone uterosacral plication (12 out of 73) compared with McCalls culdoplasty (0 out of 70) *P* = 0.000394. Inpatient stay in the uterosacral plication group was significantly shorter mean 1.8 compared to 3.6 for McCall group (*P*-Value is <0.00001). There was no significance in the perioperative complications between both groups (*P* = 0.41).

**Conclusions:**

McCalls is a superior operation to prevent PHVP compared to uterosacral plication with no difference in terms of perioperative complications.

## Background

The International Continence Society defines post-hysterectomy vault prolapse (PHVP) as descent of the vaginal cuff scar below a point that is 2 cm less than the total vaginal length above the plane of the hymen [1]. The incidence of PHVP has been reported to affect up to 43% of hysterectomies. The risk of prolapse following hysterectomy is 5.5 times more common in women whose initial hysterectomy was for pelvic organ prolapse as opposed to other reasons [1].

Preventative techniques can be used at the time of a hysterectomy to prevent PHVP. McCall culdoplasty and sacrospinous fixation can be carried out at vaginal hysterectomy [2]. Suturing the cardinal and uterosacral ligaments to the vaginal cuff at the time of abdominal or laparoscopic hysterectomy is effective in preventing post-hysterectomy vaginal prolapse [3].

Recommended management for PHVP can be largely divided into surgical and non-surgical. Methods of treatment offered depend on severity of prolapse but also takes into consideration patient wishes and expectations and suitability for surgery. Conservative management includes weight loss, treatment of constipation and avoidance of heavy lifting. Patients may also avail of physiotherapy and ring pessaries [4].

Techniques available to manage PHVP aim to ultimately suspend the vaginal vault. Approaches include vaginal, e.g. uterosacral ligament suspension, sacrospinous ligament fixation, open procedures and more recently laparoscopic, e.g. sacrocolpopexy and uterosacral plication [2, 5]. The decision-making process for managing these patients is similar to that of any prolapse, namely the response to conservative management, the effect on the quality of life and fitness for surgery [1].

## Methods

Patients were identified who underwent ‘laparoscopic hysterectomy with uterosacral plication’ and ‘vaginal hysterectomy with McCall culdoplasty’ for pelvic organ prolapse performed by two consultant gynaecologists in Northern Ireland between January 2008 and January 2014. One surgeon performed each of the described procedures.

All patients had presented with subjective symptoms of pelvic organ prolapse, and objectively, this was confirmed on objective Pelvic Organ Prolapse Quantification (POP-Q) examination.

The technique used for vaginal hysterectomy and McCall culdoplasty is described by Raymond Lee of The Mayo Clinic [6]. Following vaginal hysterectomy, one to two internal McCall sutures are placed using a zero monofilament absorbable suture. Each McCall suture is placed deeply into the left pararectal fascia then across the front of the sigmoid colon and deep into the right pararectal fascia. An external McCall suture is subsequently placed, more cephalad to the internal McCall suture. A 1-0 delayed absorbable suture is passed through the posterior vaginal wall incorporating the peritoneum. The same suture is then placed deep through the left pararectal fascia, across the sigmoid colon and deep through the right pararectal fascia. The same external McCall suture is then placed back through the vaginal wall. Depending on anterior and posterior compartment prolapse, the patients may have also undergone an anterior and/or posterior colporrhaphy. All patients underwent routine cystoscopy with indigo carmine.

In the patients undergoing uterosacral plication, following total laparoscopic hysterectomy, the ureters were re-identified. A non-absorbable, zero monofilament suture was used to place three helical sutures full thickness in each uterosacral ligament, beginning in the distal third of the ligament and incorporating the posterior vagina. The ends of the suture were tied with an extra-corporeal knot-tying technique, thus shortening the uterosacral ligaments.

Both groups of patients had their charts reviewed retrospectively and were followed up on a regional electronic care record to see if they attended anywhere in the province for subsequent pelvic organ prolapse repairs.

A total of 143 patients were identified including 73 who had undergone total laparoscopic hysterectomy and uterosacral plication and 70 who had vaginal hysterectomy and McCall culdoplasty.

Mean follow-up was 36 months (range 5–84) in the uterosacral plication group and 41 months (range 5–71) in the McCall culdoplasty group.

The notes were reviewed for parity, age, BMI, indication for surgery, the surgical procedure performed, perioperative or post-operative complications, duration of inpatient stay and findings at their 6-month post-operative review where a POP-Q was performed along with any subsequent attendances.

## Results

### Demographics

The demographics for the uterosacral plication and the McCall culdoplasty groups are summarized in Table 1.

**Table 1:** Demographics for the uterosacral plication and the McCall culdoplasty patient groups

	USP	McCall’s	*P* value
Mean age (range)	52.3 (31–72)	59 (37–82)	0.00024
Mean parity (range)	3.1 (1–6)	3.0 (1–8)	0.21
Mean BMI (range)	26.5 (16.7–41)	28.0 (20–36)	0.09

The mean parity and BMI in both groups were comparable with *P* values of 0.21 and 0.09 respectively. (*P* values were calculated using Student’s *t* test.) The mean parity in patients who underwent uterosacral plication was 3.1 compared with 3.0 in the McCall culdoplasty group. The mean BMI in patients who underwent uterosacral plication was 26.5 compared with 28.0 in the McCall culdoplasty group.

There was a statistical significance in the age difference of both groups of patients (*P* = 0.00024). The McCall patient group had a mean age of 59 (range 37–82) while the patients undergoing uterosacral plication had a mean age of 52.3 (range 31–72)

### Inpatient stay

The mean inpatient stay for patients in the laparoscopic hysterectomy and uterosacral plication group was 1.8 days (range 1–5 days). The mean inpatient stay for patients in the vaginal hysterectomy and McCall culdoplasty group was 3.6 days (range 2–7 days). There was a statistically significant difference in the duration of hospital stay in the two groups; *P* value is <0.00001 using Student’s *t* test.

### Indication

In both groups, the indication for surgery in all patients was vaginal prolapse. In patients who had objective associated anterior or posterior vaginal wall prolapse, additional procedures were carried out to address this. These procedures included anterior colporrhaphy, posterior colporrhaphy and laparoscopic paravaginal repair. Laparoscopic paravaginal repair is a procedure using a delayed absorbable suture to attach the lateral aspects of the front vaginal wall back to the arcus tendinous. It is a procedure used to address anterior lateral vaginal wall defects.

In the patients undergoing vaginal hysterectomy and McCall culdoplasty, four patients also complained of heavy menstrual bleeding. In the patients undergoing laparoscopic hysterectomy and uterosacral ligament plication, three patients also complained of heavy menstrual bleeding.

### Procedure

Details of the procedures performed for utero-vaginal prolapse are summarized in Tables 2 and 3.

**Table 2:** Details of laparoscopic procedure

Procedure	Number of patients
Total laparoscopic hysterectomy (±BSO) and uterosacral plication	32
Total laparoscopic hysterectomy (±BSO) and uterosacral plication and posterior colporrhaphy	21
Total laparoscopic hysterectomy (±BSO) and uterosacral plication and laparoscopic paravaginal repair	9
Total laparoscopic hysterectomy (±BSO) and uterosacral plication and posterior colporrhaphy and laparoscopic paravaginal repair	5
Total laparoscopic hysterectomy (±BSO) and uterosacral plication and anterior colporrhaphy	5
Total laparoscopic hysterectomy (±BSO) and uterosacral plication and anterior colporrhaphy and posterior colporrhaphy	1
Total	73

**Table 3:** Details of vaginal procedure

Procedure	Number of patients
Vaginal hysterectomy (±BSO) and McCall culdoplasty and anterior colporrhaphy and posterior colporrhaphy	60
Vaginal hysterectomy (±BSO) and McCall culdoplasty and anterior colporrhaphy	4
Vaginal hysterectomy (±BSO) and McCall culdoplasty	3
Vaginal hysterectomy (±BSO) and McCall culdoplasty and posterior colporrhaphy	3
Total	70

### Complications

Seventeen patients in total had reported complications. This included ten patients in the McCall culdoplasty group and seven patients in the uterosacral plication group. See Tables 4 and 5 for details.

**Table 4:** Complications in uterosacral plication patient group

Complication	Operation	Management
Wound infection	Laparoscopic hysterectomy and uterosacral plication and posterior colporrhaphy	Oral antibiotics
Wound infection	Laparoscopic hysterectomy and uterosacral plication	Oral antibiotics
Wound infection	Laparoscopic hysterectomy and uterosacral plication	IV antibiotics
Urinary retention	Laparoscopic hysterectomy and uterosacral plication and posterior colporrhaphy and paravaginal repair	Conservative—ISC
Urinary retention	Laparoscopic hysterectomy and uterosacral plication and paravaginal repair	Revision of sutures at bladder neck following paravaginal repair
Vault haematoma	Laparoscopic hysterectomy and uterosacral plication	IV antibiotics
Port site herniation	Laparoscopic hysterectomy and uterosacral plication	Surgically managed

**Table 5:** Complications in the McCall culdoplasty patient group

Complication	Operation	Management
Urinary retention	Vaginal hysterectomy and McCall culdoplasty and anterior colporrhaphy and posterior colporrhaphy	Conservative—ISC
Urinary retention	Vaginal hysterectomy and McCall culdoplasty and anterior colporrhaphy and posterior colporrhaphy	Conservative—ISC
Urinary retention	Vaginal hysterectomy and McCall culdoplasty and anterior colporrhaphy and posterior colporrhaphy	Conservative—ISC
Urinary retention	Vaginal hysterectomy and McCall culdoplasty and anterior colporrhaphy and posterior colporrhaphy	Conservative—ISC
Urinary retention	Vaginal hysterectomy and McCall culdoplasty and anterior colporrhaphy and posterior colporrhaphy	Conservative—ISC
Urinary retention	Vaginal hysterectomy and McCall culdoplasty and anterior colporrhaphy and posterior colporrhaphy	Conservative—ISC
Post-operative bleeding	Vaginal hysterectomy and McCall culdoplasty and anterior colporrhaphy and posterior colporrhaphy	Return to theatre; laparotomy
Post-operative bleeding	Vaginal hysterectomy and McCall culdoplasty and anterior colporrhaphy and posterior colporrhaphy	Return to theatre; laparotomy
Post-operative dyspareunia	Vaginal hysterectomy and McCall culdoplasty and anterior colporrhaphy and posterior colporrhaphy	Blair Bell/Fenton’s procedure
Ureteric obstruction seen at cystoscopy	Vaginal hysterectomy and McCall culdoplasty and anterior colporrhaphy and posterior colporrhaphy	Release of McCall culdoplasty intraoperatively

There was no significance in the perioperative complications between both groups (*P* = 0.41)

In the patients undergoing laparoscopic hysterectomy and uterosacral plication, three patients require antibiotics for port site wound infections. Two patients had post-operative urinary retention, one that was managed conservatively and one that required release of sutures at the bladder neck following paravaginal repair. One patient re-attended with port site herniation, 2 weeks after surgery, that required surgical management. One patient had a vault haematoma, which was managed conservatively with antibiotics.

One patient undergoing McCall culdoplasty required intraoperative release of the McCall due to evidence of ureteric obstruction at routine cystoscopy performed during the procedure. Six patients in the McCall culdoplasty group had post-operative urinary retention. All of these were successfully managed conservatively with a period of intermittent self-catheterization. Two patients returned to theatre for a laparotomy for post-operative intra-abdominal bleeding in the first 24 h post-operatively. One patient required a subsequent Blair Bell (Fenton’s) procedure for post-operative dyspareunia which failed to respond to conservative measures.

### Post-operative findings

Mean follow-up time was 36 months in the uterosacral plication group and 41 months in the McCall culdoplasty group. All patients were assessed 6 months post-operatively where subjective and objective (POP-Q) assessments of subsequent prolapse symptoms were ascertained by the same two surgeons.

#### Uterosacral plication

In the uterosacral plication group, 53 out of 73 (72.6%) patients had no further pelvic organ prolapse.

Twelve patients (16.4%) have had PHVP. Eight patients have opted for surgical repair. Of the eight patients undergoing surgical repair for PHVP, four patients had a subsequent laparoscopic sacrocolpopexy, one patient had a laparoscopic sacrocolpopexy that was converted to an open procedure intraoperatively due to dense adhesions, one patient had a sacrospinous ligament fixation and two patients had repeat uterosacral ligament plications performed. Four patients opted for insertion of vaginal pessary. See Table 6.

**Table 6:** Vaginal vault prolapse following laparoscopic hysterectomy and uterosacral plication

Original operation	Repair of PHVP
Laparoscopic hysterectomy and uterosacral plication	Laparoscopic sacrocolpopexy
Laparoscopic hysterectomy and uterosacral plication and paravaginal repair	Laparoscopic sacrocolpopexy
Laparoscopic hysterectomy and uterosacral plication	Laparoscopic sacrocolpopexy
Laparoscopic hysterectomy and uterosacral plication and paravaginal repair	Laparoscopic sacrocolpopexy
Laparoscopic hysterectomy and uterosacral plication and paravaginal repair	Laparoscopic sacrocolpopexy converted to open sacrospinous ligament fixation
Laparoscopic hysterectomy and uterosacral plication	Vaginal sacrospinous ligament fixation
Laparoscopic hysterectomy and uterosacral plication	Laparoscopic uterosacral ligament plication
Laparoscopic hysterectomy and uterosacral plication and paravaginal repair	Laparoscopic uterosacral ligament plication
Laparoscopic hysterectomy and uterosacral plication and anterior colporrhaphy	Pessary
Laparoscopic hysterectomy and uterosacral plication	Pessary
Laparoscopic hysterectomy and uterosacral plication and posterior colporrhaphy	Pessary
Laparoscopic hysterectomy and uterosacral plication	Pessary

Seven patients (9.5%) have had de novo anterior compartment prolapse. Four patients (5.4%) had recurrence of anterior wall prolapse. Seven patients opted for surgical repair, two patients opted for vaginal pessary insertion and two patients have chosen conservative management. See Table 7.

**Table 7:** Anterior compartment prolapse following laparoscopic hysterectomy and uterosacral plication

Original operation	Management
Laparoscopic hysterectomy and uterosacral plication and posterior colporrhaphy	Anterior colporrhaphy
Laparoscopic hysterectomy and uterosacral plication	Anterior colporrhaphy
Laparoscopic hysterectomy and uterosacral plication	Anterior colporrhaphy
Laparoscopic hysterectomy and uterosacral plication and anterior colporrhaphy	Anterior colporrhaphy
Laparoscopic hysterectomy and uterosacral plication and paravaginal repair	Laparoscopic paravaginal repair
Laparoscopic hysterectomy and uterosacral plication	Laparoscopic paravaginal repair
Laparoscopic hysterectomy and uterosacral plication and anterior colporrhaphy	Vaginal Elevate mesh
Laparoscopic hysterectomy and uterosacral plication	Conservative—no treatment
Laparoscopic hysterectomy and uterosacral plication and paravaginal repair	Conservative—no treatment
Laparoscopic hysterectomy and uterosacral plication	Pessary
Laparoscopic hysterectomy and uterosacral plication	Pessary

Four patients (5.4%) have had de novo posterior compartment prolapse; three have opted for surgical repair

#### McCall culdoplasty

In the McCall culdoplasty group, there have been no patients with PHVP. Four patients have represented with posterior compartment prolapse (Table 8), and two patients have represented with anterior compartment prolapse. Two of these patients have required surgical management for their symptoms. One patient underwent a subsequent anterior colporrhaphy, and one patient has undergone a subsequent posterior colporrhaphy. See Tables 9 and 10.

**Table 8:** Posterior compartment prolapse following laparoscopic hysterectomy and uterosacral plication

Original operation	Management
Laparoscopic hysterectomy and uterosacral plication	Posterior colporrhaphy
Laparoscopic hysterectomy and uterosacral plication and paravaginal repair	Posterior colporrhaphy
Laparoscopic hysterectomy and uterosacral plication	Posterior colporrhaphy
Laparoscopic hysterectomy and uterosacral plication and anterior colporrhaphy	Conservative—no treatment

**Table 9:** Anterior compartment prolapse following vaginal hysterectomy and McCall culdoplasty

Original operation	Management
Vaginal hysterectomy and McCall culdoplasty and anterior colporrhaphy and posterior colporrhaphy	Anterior colporrhaphy
Vaginal hysterectomy and McCall culdoplasty and anterior colporrhaphy and posterior colporrhaphy	Conservative

**Table 10:** Posterior compartment prolapse following vaginal hysterectomy and McCall culdoplasty

Original operation	Management
Vaginal hysterectomy and McCall culdoplasty and anterior colporrhaphy and posterior colporrhaphy	Posterior colporrhaphy
Vaginal hysterectomy and McCall culdoplasty and anterior colporrhaphy and posterior colporrhaphy	Conservative
Vaginal hysterectomy and McCall culdoplasty and anterior colporrhaphy and posterior colporrhaphy	Conservative
Vaginal hysterectomy and McCall culdoplasty and anterior colporrhaphy and posterior colporrhaphy	Conservative

## Discussion and conclusions

The aetiology of PHVP is multifactorial; however, damage to the level one supports of the vagina during hysterectomy are thought to be a major contributing factor. The risk of this is thought to be greatest when the hysterectomy is performed for the indication of pelvic organ prolapse [7].

There are very few studies comparing vaginal McCall culdoplasty to laparoscopic uterosacral plication for prevention of subsequent prolapse.

In 1957, McCall described attaching the uterosacral ligaments to the posterior vaginal cuff and the cul de sac peritoneum in order to close off the cul de sac and prevent subsequent prolapse [8].

Uterosacral plication does not obliterate the cul de sac. It involves placing sutures distally on the uterosacral ligaments and tying them in the midline under tension away from their attachment into the vagina. The support this provides for the vagina has so far been unclear [9]. One of the theoretical advantages of laparoscopic over vaginal technique is the ability to identify the ureters, thus reducing the chance of inadvertent ureteric injury. Our study shows that in trained hands and with the prudent employment of indigo carmine and routine cystoscopy, the rate of ureteric injury is not significantly higher in the vaginal McCall group.

This study has retrospectively evaluated the McCall culdoplasty and the laparoscopic uterosacral plication when performed alongside hysterectomies in order to prevent PHVP. It has found them comparable in terms of complications encountered. Laparoscopic uterosacral plication has a statistically significant shorter hospital admission; however, McCall culdoplasty has proven to be superior to laparoscopic uterosacral plication in terms of patients representing with subsequent pelvic organ prolapse.

While both groups had a low rate of PHVP, in this study, McCall culdoplasty was a more successful operation compared to uterosacral plication with no difference in terms of perioperative complications.

## Abbreviations

BMI: Body mass index
BSO: Bilateral salpingo-oophorectomy
ISC: Intermittent self-catheterization
PHVP: Post-hysterectomy vault prolapse
POP-Q: Pelvic Organ Prolapse Quantification
